# Extracellular vesicles as modulators of cancer metabolism and microenvironment

**DOI:** 10.20517/evcna.2025.151

**Published:** 2026-01-19

**Authors:** Veronica Feltri, Alessio Sanfilippo, Roberta Tasso, Maria Chiara Ciferri

**Affiliations:** ^1^Department of Experimental Medicine, University of Genoa, Genoa 16132, Italy.; ^2^IRCCS Azienda Ospedaliera Metropolitana, Genova 16132, Italy.; ^#^These authors contributed equally to this work.

**Keywords:** Cancer microenvironment, cancer metabolism, tumor-derived extracellular vesicles

## Abstract

The existence of small vesicles released by cells into the extracellular space was first documented over 40 years ago. These nanoparticles, now recognized as extracellular vesicles (EVs), were originally defined as “*cellular dust*” reflecting the early belief that their primary function was to dispose of cellular waste. Nowadays, it is widely acknowledged that EVs make a fundamental contribution to intercellular communication, being capable of transporting biologically active molecules, including proteins and nucleic acids, which regulate both physiological and pathological processes. Their involvement in various diseases, particularly cancer, has been well documented. EVs influence tumor development, progression, and therapeutic response, and have therefore been considered potential diagnostic and prognostic biomarkers. In this review, we focus on the contribution of EVs in modulating tumor cell metabolism and the tumor microenvironment. Specifically, we describe how EVs promote angiogenesis, induce the transformation of fibroblasts into cancer-associated fibroblasts, and influence extracellular matrix remodeling. Additionally, we explore their contribution to the reprogramming of tumor metabolism, including glycolytic, lipid, and amino acid pathways. We provide an in-depth overview of the key molecules carried by EVs that contribute to these pro-tumorigenic effects and of the underlying mechanisms involved.

## INTRODUCTION

### Tumor-induced alterations of the cell microenvironment

For many years, cancer research has primarily focused on tumor cells, overlooking the surrounding microenvironment that has indeed been demonstrated to play a pivotal role in cancer development and dissemination^[1]^. Tumor microenvironment (TME) comprises all cellular and non-cellular components encompassing the tumor, which include stromal, endothelial, and immune cells, as well as the extracellular matrix (ECM) and various signaling factors, such as cytokines, chemokines, and growth factors^[2]^. It has been shown that these constituents, although not being strictly part of the tumor mass, are effectively involved in many processes fostering tumor progression and colonization of metastatic niches^[3]^. In the early stages of cancer, the complex microenvironment largely maintains physiological behavior, attempting to restore the homeostasis altered by tumor growth; however, over time, it becomes subverted by pathological mechanisms^[4]^. One of the most characteristic processes witnessing this subjugation is angiogenesis. Cancer cells can stimulate the abnormal proliferation of existing blood vessels to secure oxygen and nutrients required for their growth. This happens thanks to the release of pro-angiogenic factors, notably vascular endothelial growth factor (VEGF), but also platelet-derived growth factor (PDGF) and angiopoietin (ANG)^[5]^. Moreover, both endothelial cells of blood vessels and lymphatic vasculature acquire peculiar features in the TME, giving birth to the so-called enhanced permeation and retention (EPR) effect^[6]^. This phenomenon, often exploited in passive targeting nanomedicine approaches, is related to the presence of a leaky vasculature and an impaired lymphatic drainage at the tumor site^[7]^. Similarly, tumor-surrounding fibroblasts typically undergo phenotypic changes, becoming recruited and activated as cancer-associated fibroblasts (CAFs). These cells support tumor progression by secreting growth factors and inducing ECM remodeling^[8]^. The latter process involves changes in the composition, stiffness, and structure of the complex and dynamic network of proteins (mainly collagen, elastin, fibronectin, and laminin) and proteoglycans forming the cell-hosting matrix. Increased collagen deposition and cross-linking, which support tumor growth, together with proteolytic matrix rearrangement, which facilitates cancer cell migration, are two of the main ECM modifications typically observed in the TME^[9]^. In addition, epithelial cells, under the influence of factors such as interleukin (IL)-1, IL-6, and tumor necrosis factor alpha (TNF-α), can undergo a reversal of epithelial-to-mesenchymal transition (EMT), reacquiring a mesenchymal-like state with enhanced migratory potential^[10]^. Significant changes also occur in immune cells, including T cells, natural killer (NK) cells, dendritic cells, and macrophages. Depending on their activation state, these cells can exhibit either anti- or pro-tumor activities: some immune components contribute to tumor-suppressive responses, while cancer cells can exploit mechanisms that allow them to evade immune surveillance^[11]^. Examples of such pro-tumor involvement include the recruitment of immunosuppressive T regulatory cells (Tregs) and myeloid-derived suppressor cells (MDSCs), as well as the formation of tumor-associated macrophages (TAMs). In addition, the expression of specific immune checkpoint proteins, such as programmed cell death receptor ligand 1 (PD-L1), can inhibit immune cell activity by interacting with their receptors^[12]^.

### Cancer metabolism: bioenergetic and biosynthetic pathways

Tumor-related modifications of cell metabolism are included among the current “Hallmarks of Cancer”^[13]^ and recognized as fundamental adaptations that allow tumors to survive, proliferate and adjust to the hostile environment^[14]^. During the neoplastic transformation, tumor cells undergo a deep metabolic remodeling by which the main bioenergetic and biosynthetic pathways are redefined to meet the great demand of energy and molecules required for tumor growth and survival.

In healthy cells, energy demand is primarily sustained through the tricarboxylic acid (TCA) cycle and oxidative phosphorylation (OXPHOS), which can jointly generate up to 38 molecules of adenosine triphosphate (ATP) per glucose^[15]^. Glycolysis, which converts glucose into pyruvate, produces a smaller fraction of ATP under aerobic conditions, but can provide energy anaerobically when oxygen is limited^[14]^. However, even in the presence of oxygen^[16]^, tumor cells preferentially metabolize glucose through glycolysis, a less efficient but faster process that produces pyruvate and only two molecules of ATP per glucose molecule^[17]^. This phenomenon, also known as “Warburg Effect”, has been deeply studied, although its mechanistic basis remains only partially understood^[14]^. Also, lactate, produced by the fermentation of pyruvate and once considered a waste product, is now known as a key metabolite involved in tumor development^[18]^. Beyond contributing to the acidification of the TME and promoting angiogenesis, lactate can be taken up by normoxic tumor cells, where it is converted back into pyruvate by lactate dehydrogenase B (LDH-B) and subsequently oxidized through the TCA cycle^[19]^. Rather than glycolytic alterations, lipid metabolism has recently gained increasing attention, as dysregulated lipid metabolism in the TME is closely linked to cell proliferation, metastasis, and invasion^[20]^. Lipids are fundamental molecules involved in maintaining cell structure, providing energy, and regulating cellular signaling pathways. Beyond these physiological functions, their contribution to tumor progression has also been well documented. A typical example is represented by fatty acids (FAs), integral components of phospholipids, sphingolipids, diacylglycerols, and triacylglycerols^[21]^. These components contribute to maintaining membrane composition and fluidity, while also serving as secondary messengers in numerous signaling pathways^[20]^. Their involvement in carcinogenesis is now well established, as they can fuel tumor growth, modulate membrane-associated signaling, and influence processes such as proliferation, migration and resistance to therapy^[22]^. Phosphatidylserine (PS), a phospholipid component of the cellular membrane derived from FAs, is well known for its immunosuppressive role in several cancers, including colon cancer^[23]^. Similarly, cholesterol plays a crucial role in sustaining tumor proliferation. Tumor cells often accumulate cholesterol to maintain a readily available reservoir, a process associated with the overexpression of low-density lipoprotein (LDL) receptors, particularly observed in breast^[24]^ and colon^[25]^ cancers.

Moreover, alterations of amino acid metabolism, such as glutamine (Gln) addiction, have been described as an emerging characteristic of several types of cancer^[26]^. Gln is the most abundant amino acid in plasma, and its importance lies in its ability to act as both a nitrogen and carbon donor^[27]^. In addition, it contributes to the production of lipids and glutathione^[28]^. Gln is converted into glutamate by glutaminase (GLS) and then dehydrogenized by glutamate dehydrogenase (GLUD1) to produce α-ketoglutarate (α-KG), a key intermediate of the TCA cycle. This reaction not only sustains anaplerosis, but also generates nicotinamide adenine dinucleotide phosphate (NADPH), a crucial cofactor for maintaining redox balance and promoting cell survival under oxidative stress^[29]^. For these reasons, Gln has been recognized as a vital metabolite for tumor survival. In cancer, there is a significant increase in the demand for Gln by tumor cells. As a result, maintaining high levels of glutamine becomes crucial^[30]^. Under glutamine starvation, cancer cells activate adaptive mechanisms including autophagy and micropinocytosis, as observed in pancreatic ductal adenocarcinoma (PDAC)^[31]^. These processes enable the recycling or scavenging of extracellular metabolites by which the tumor cells can compensate for the high glutamine demand. Recent evidence has shown that, in the absence of glutamine, mesenchymal cells and CAFs are able to invade nearby tissues by following a glutamine gradient, underlying its importance in metastasis and tumor invasion^[32]^. The metabolic interplay between glutamine and asparagine provides an adaptive mechanism that supports tumor cell survival under nutrient stress^[33]^. In fact, asparagine - a non-essential amino acid that plays a key role in the production of molecules such as glucose, proteins, lipids and nucleotides^[34]^ - is synthesized by asparaginase (ASNS), an enzyme using ATP and glutamine as a source of nitrogen^[35]^. Some studies have highlighted that resistance to ASNS is often correlated with increased glutamine synthetase (GS) activity^[36]^. Moreover, asparagine has been identified as a crucial metabolite for tumor survival under glutamine deprivation. Recently, Zhang *et al.* reported that among various amino acids considered, asparagine was the only one that prevented glutamine deprivation-induced apoptosis, emphasizing the strong metabolic interdependence between these two amino acids^[37]^. Likewise, branched amino acids (BCAAs) and their metabolic pathway are included among cancer-associated alterations. BCAAs represent a class of essential amino acids (leucine, isoleucine, and valine) that cannot be synthesized by the organism but need to be assimilated from the diet^[38]^. The metabolism of these amino acids depends on two enzymes: branched-chain aminotransferases 1 and 2 (BCAT-1 and BCAT-2), which convert BCAAs into their corresponding α-ketoacids (BCKA), thus producing α-KG and glutamate. These BCKAs are subsequently converted into branched-chain acyl coenzyme A (acyl-CoA) esters, generating 1,4-dihydronicotinamide adenine dinucleotide (NADH) and contributing to cellular energy production. It has been demonstrated that the cytoplasmic localization of BCAT-1 correlates with cancer progression, thus making this enzyme a potential diagnostic marker^[39-42]^. Contrary to isoform 1, BCAT-2 is predominantly expressed in the mitochondria of metabolically active tissues such as the liver and spleen. BCAT-2 has been particularly studied in melanoma and PDAC, where it has been shown to be responsible for tumor progression^[43,44]^. The development of inhibitors targeting BCAT1 and BCAT2 represents a promising therapeutic strategy based on inhibition of BCAA metabolism and subsequent impairment of tumor cell energy supply and biosynthetic pathways, thereby suppressing cell growth and proliferation.

Therefore, both the microenvironment and metabolism^[45-48]^ are two key determinants influencing how tumors grow, spread, evade the immune system, and develop treatment resistance^[49]^. Within this complex network, tumor-derived extracellular vesicles (T-EVs) play a crucial role as intercellular messengers able to transfer neoplastic traits to other cells and influence their behavior^[50]^.

### Extracellular vesicles as intercellular communicators

Extracellular vesicles (EVs) are a heterogeneous group of nano- to micro-sized particles delimited by a phospholipid bilayer that can be released by any cell type to transport information and facilitate cell-to-cell communication^[51,52]^. They are characterized by a complex composition that includes specific surface proteins and lipids, as well as cargo enriched in various molecules, such as RNAs, cytosolic proteins, and signaling molecules^[53]^. Biogenesis, size, or physical characteristics can be used to define the different EV subtypes and classify them accordingly^[52]^. Depending on whether they originate from the endosomal system or the plasma membrane, EVs can be classified as exosomes or ectosomes (historically known as “microvesicles”)^[52]^. Specifically, after endocytosis, the early endosome membrane undergoes inward budding forming intraluminal vesicles (ILVs). During maturation and accumulation of these vesicles, the endosome becomes a multivesicular body (MVB)^[54,55]^. ILV fate depends on the subsequent trafficking of the MVB: it may fuse with lysosomes, leading to degradation of its content, or with the plasma membrane, resulting in the release of ILVs into the extracellular space, where they are referred to as exosomes^[54-58]^. In contrast, ectosomes are released directly into the extracellular space via outward protrusions or budding from the plasma membrane^[57-59]^. The diameter, which can be above or below 200 nm, determines whether EVs are classified as “large” or “small”. Exosomes are generally smaller than 200 nm, whereas ectosomes exhibit a broader size distribution, including sizes comparable to exosomes^[52]^. Specific cellular processes, such as migration or programmed cell death, can produce EVs denoted by specialized terms, including “migrasomes”^[60]^ or “apoptotic bodies”^[61]^. EV molecular cargo often reflects the state of the cells from which they originate, offering a window into tissue-specific activity^[52,62,63]^. The tiny packages they carry and their composition often contain molecules directed toward specific target cells and can be modified or adapted depending on biological changes and the stage of a particular alteration or disease^[59]^. For these reasons, EVs have become useful not only in providing insights into cellular physiological mechanisms but also for studying disease onset and possibly tracking its progression over time. In this sense, the potential clinical application of natural nanoparticles as disease biomarkers in the screening, diagnosis, prognosis and monitoring of cancer have been highly discussed^[64-68]^; their involvement has been successfully demonstrated in different tumor types such as breast^[69,70]^, colorectal^[71-73]^, lung^[74-76]^ and pancreatic cancer^[77,78]^. These findings highlight how EVs can influence tumor behavior in diverse ways and that their cargo can promote cell proliferation, enhance the aggressiveness of cancer cells, or even contribute to treatment resistance. Interestingly, some therapies may induce cancer cells to release vesicles that transfer drug resistance to neighboring cells^[79]^.

Considering that EVs have shown to modulate TME and metabolism, this review explores their role in shaping these cancer-related processes.

## EV CONTRIBUTION TO TME CROSSTALK

**Figure 1:**
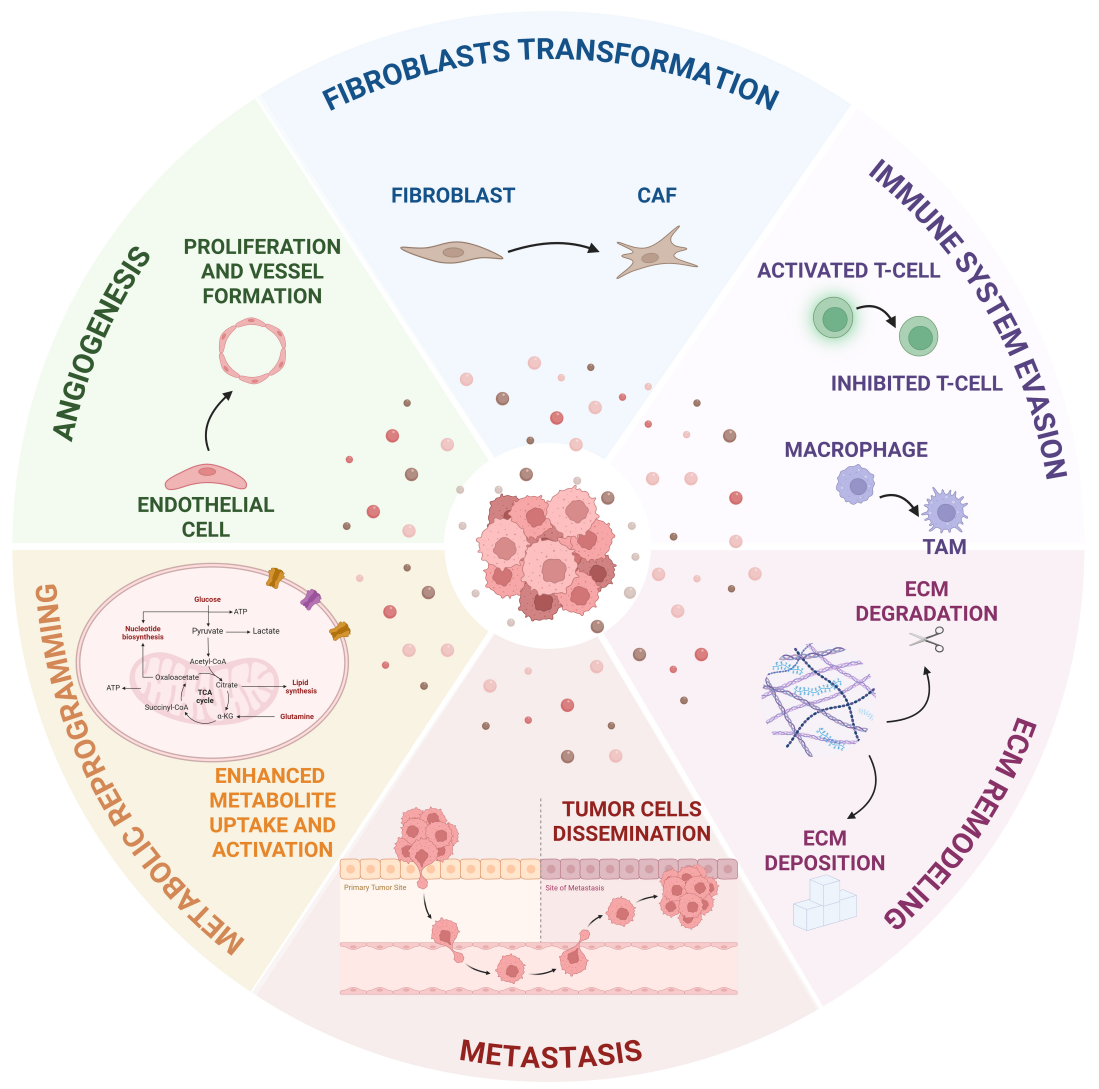
Schematic illustrating the various mechanisms through which T-EVs modulate the tumor microenvironment and tumor metabolism. T-EVs promote angiogenesis, fibroblast transformation into CAFs, immunosuppression, ECM remodeling, metastasis formation, and metabolic reprogramming by transferring a wide range of tumor-derived molecules. Created with Biorender. Sanfilippo, A. (2026) https://BioRender.com/mojdgw1. EVs: Extracellular vesicles; T-EVs: tumor-derived extracellular vesicles; CAFs: cancer-associated fibroblasts; ECM: extracellular matrix; TAM: tumor-associated macrophage; ATP: adenosine triphosphate; TCA: tricarboxylic acid; CoA: coenzyme A; α-KG: alpha-ketoglutarate.

T-EVs can transport neoplastic traits to both nearby and distant cells, affecting their behavior and influencing the surrounding microenvironment toward the acquisition of cancer-related modifications^[80]^*.* The discussion of the following paragraph will focus on EV contribution in transforming the TME by evaluating their involvement in different related mechanisms including angiogenesis promotion, fibroblast transformation, immune system evasion, ECM remodeling, and metastasis formation [Figure 1].

### Angiogenesis promotion

Angiogenesis is fundamental to supplying tumor cells with oxygen and nutrients necessary for their growth. The creation of new blood vessels from existing ones is triggered by the release of soluble growth factors capable of recruiting endothelial cells in the tumor area and inducing their proliferation. Interestingly, it has been observed that this process is often promoted by T-EVs through diverse signaling pathways^[81]^ [Table 1]. Indeed, T-EVs have been reported to transport various pro-angiogenic molecules, including non-coding RNAs (ncRNAs), such as circular RNAs (circRNAs), long ncRNAs (lncRNAs), and microRNAs (miRNAs), as well as proteins, with VEGF being one of the most well-known and extensively investigated^[82]^. ncRNAs support angiogenesis by regulating gene expression, whereas proteins do so by interacting with their receptors. Besides VEGF, several other proteins play significant roles, including PDGF, epidermal growth factor (EGF), fibroblast growth factor (FGF), transforming growth factor (TGF), matrix metalloproteinases (MMPs), and ANGs^[83]^. T-EVs can mediate angiogenesis either by directly delivering pro-angiogenic molecules to endothelial cells, thus enhancing their migration and vessel formation, or by inducing the transformation of fibroblasts or MSCs into CAFs, as well as macrophages into TAMs, both known as key promoters of angiogenesis^[84,85]^. For example, Feng *et al.* reported that EVs derived from breast cancer cells could activate VEGF receptors (VEGFRs) on endothelial cells thanks to a 90 kDa form of VEGF associated with their surface^[86]^. In the same context, Kalfon *et al.* found that gastric cancer-derived EVs containing angiopoietin-2 (ANG2) could induce angiogenesis through the phosphatidylinositol 3-kinase (PI3K)/AKT serine/threonine kinase signaling pathway^[87]^. Similarly, another study conducted by Huang *et al.* proved that tumor perivascular cell-derived EVs could exert the same effect via the growth arrest–specific protein 6 (GAS6)/AXL receptor tyrosine kinase (AXL) signaling cascade^[88]^. Additionally, Yan *et al.* highlighted that EVs derived from TAMs can transfer miR-21-5p to endothelial cells, thereby promoting angiogenesis by regulating the Yes-associated protein 1 (YAP1)/hypoxia-inducible factor-1 alpha signaling axis (HIF-1α) axis in head and neck squamous cell carcinoma^[84]^.

**Table 1:** Molecular mechanisms by which tumor-derived EVs promote angiogenesis

**EV source**	**Functional cargo components**	**Mechanism of action**	**Ref.**
Breast cancer cells (MDA-MB-231 cell line)	VEGF90K	Activation of VEGFR on endothelial cells	[86]
Gastric cancer cells (AGS and SNU-16 cell lines)	ANG2	Activation of the PI3K/AKT transduction pathway in endothelial cells	[87]
Tumor perivascular cells	GAS6	Activation of the AXL pathway in endothelial cells	[88]
THP-1-derived TAMs	miR-21-5p	Upregulation of the YAP1/HIF-1α axis in endothelial cells	[84]

EVs: Extracellular vesicles; VEGF90K: 90kDa form of vascular endothelial growth factor; VEGFR: vascular endothelial growth factor receptor; ANG2: angiopoietin-2; PI3K/AKT: phosphoinositide 3-kinase/protein kinase B; GAS6: growth arrest-specific 6; AXL: AXL receptor tyrosine kinase; TAMs: tumor-associated macrophages; YAP1/HIF-1α: yes-associated protein 1/hypoxia-inducible factor 1, α subunit.

### Fibroblast transformation

Fibroblasts are important constituents of the stroma, with their main function being the synthesis of all major components of the ECM, thereby providing the basal scaffold for tissues. In tumors, they often acquire a malignant-like phenotype, being transformed into CAFs. CAFs display several pro-oncogenic activities, contributing to angiogenesis, immune evasion, ECM remodeling, metabolic reprogramming, and metastasis formation^[89]^. They can be generated not only from fibroblasts, but also from MSCs. This process is driven by different signaling molecules, both nucleic acids and proteins, often carried by T-EVs [Table 2]. Some studies have highlighted how CAFs can be activated by oncogenic proteins packaged into T-EVs, such as latent membrane protein-1 (LMP1)^[90]^ and gain-of-function p53 mutants^[91]^. Other studies have investigated the correlation between specific ncRNAs contained in T-EVs and the conversion of fibroblasts to a cancer-associated phenotype. For instance, Zhou *et al.* demonstrated that melanoma cell-derived exosomal miR-155-5p could promote a pro-angiogenic switch of fibroblasts via the suppressor of cytokine signaling-1 (SOCS1)/Janus kinase-2 (JAK2)/signal transducer and activator of transcription-3 (STAT3) signaling pathway^[92]^. Similarly, it has been shown that miR-21 can confer fibroblasts an invasive potential thanks to increased expression of MMP2 and MMP9^[93]^; additionally, miR-155 and miR-210 can modulate stromal cells’ metabolism oncogenically^[94]^. Other research studies have shown that miR-1290, packaged into high-metastatic lung cancer cell-derived EVs, could activate normal fibroblasts into CAFs through the metallothionein-1G–AKT serine (MT1G)/threonine kinase regulatory (AKT) pathway^[95]^. Similarly, miR-92b-3p from melanoma cell-derived EVs exerts the same effect by downregulating phosphatase and tensin homolog (PTEN) and subsequently activating the PI3K/AKT pathway^[96]^. Moreover, circular RNA originating from the Eps15 homology domain-containing 2 (EHD2), present in EVs from renal cell carcinoma, has also been found to promote metastasis by converting fibroblasts to CAFs^[97]^.

**Table 2:** Molecular mechanisms by which tumor-derived EVs induce fibroblasts transformation into cancer-associated fibroblasts

**EV source**	**Functional cargo components**	**Mechanism of action**	**Ref.**
Nasopharyngeal carcinoma (CNEI-LMP1 cell line)	LMP1	Activation of NF-kB pathway in fibroblasts	[90]
Colorectal cancer cells (TP53 HT-29 mutant cell line)	Gain-of-function p53	Activation of NRF2-mediated pathways in fibroblasts	[91]
Melanoma cells (A375, B16 and B16-F10 cell lines)	miR-155-5p	Downregulation of SOCS1 with activation of JAK2/STAT3 signaling pathway in fibroblasts	[92]
Melanoma cells (B16-F10 cell line)	miR-21	Increase of MMP2 and MMP9 expression in fibroblasts	[93]
Melanoma cells (BRAF WT - 1770-Her4, 2183-Her4, 1300-mel and HMCB - and BRAF V600E mutant - 526-mel, 888-mel and Hs 294 T- cell lines)	miR-155 miR-210	Increase of glycolysis and decrease of oxidative phosphorylation in fibroblasts	[94]
High-metastatic lung cancer cells (95D cell line)	miR-1290	Modulation of MT1G/AKT pathway in fibroblasts	[95]
Melanoma cells (BLM and MV3 cell lines)	miR-92b-3p	Downregulation of PTEN with activation of PI3K/AKT pathway in fibroblasts	[96]
Renal cell carcinoma cells (OSRC-2, 786-O, Caki-1, 769 P cell lines ) and patient serum	circEHD2	Package of circEHD2 into T-EVs and delivery to fibroblasts	[97]

EVs: Extracellular vesicles; LMP1: latent membrane protein 1; NF-kB: nuclear factor kappa B; NRF2: nuclear factor erythroid 2-related factor 2; SOCS1: suppressor of cytokine signaling 1; JAK2/STAT3: Janus kinase 2/signal transducer and activator of transcription 3; MMP2: matrix metalloproteinase 2; MMP9: matrix metalloproteinase 9; MT1G/AKT: metallothionein 1G/ protein kinase B; PTEN: phosphatase and tensin homolog; PI3K/AKT: phosphoinositide 3-kinase/protein kinase B; T-EVs: tumor-derived EVs.

### Immune system evasion

Cancer cells have the ability to evade immune surveillance through different mechanisms, such as the recruitment of immunosuppressive Tregs and MDSCs, macrophage polarization towards an alternatively activated (M2) pro-tumor phenotype, and the inhibition of T-cells and NK cells that generally display an anti-tumor behavior^[12]^. Malignant cells use specific immune checkpoint molecules, such as PD-L1, to “switch off” T-cell responses. PD-L1 is the ligand for programmed death 1 (PD-1) receptor, expressed on the surface of diverse immune cells, especially CD8+ T-cells. The interaction between PD-L1 and its receptor causes immune cell inactivation^[98]^. Interestingly, it has been found that most of these evasion mechanisms are T-EVs-mediated [Table 3]. Many studies have reported the presence of PD-L1 in EVs derived from different tumor types^[99,100]^. Another mechanism of T-EV-mediated immunosuppression involves an increase in extracellular adenosine levels. Adenosine can be directly released from these vesicles^[101]^; however, the presence of the ectonucleotidases CD39 and CD73 on their surface also contributes to ATP conversion into adenosine^[102]^. This nucleoside, by binding to its receptors on immune cells, promotes cAMP synthesis, thereby leading to immune inhibition^[81]^. It has been reported that some T-EVs are also enriched in molecules able to induce the M2 phenotype of TAMs, hence creating an immunosuppressive pro-metastatic environment. For example, Gerloff *et al.* showed that melanoma-derived exosomal miR-125b-5p could convert macrophages into TAMs by targeting the lysosomal acid lipase A (LIPA)^[103]^. In the same context, Yao *et al.* found that circular RNA derived from ATPase phospholipid transporting 9A (circATP9A) contained in non-small cell lung cancer-derived EVs could promote macrophage M2 polarization^[104]^; similarly, Zhang *et al.* described that complement C3 of renal cell carcinoma-derived EVs, besides favoring the M2 phenotype, could also recruit polymorphonuclear MDSCs^[105]^.

**Table 3:** Molecular mechanisms by which tumor-derived EVs contribute to immune system evasion

**EV source**	**Functional cargo components**	**Mechanism of action**	**Ref.**
Oral squamous cell carcinoma cells (SAS and TW2.6 cell lines) and patient blood	PD-L1	Interaction with PD-1 on T-cells	[99]
Melanoma cells (MEL624, PD-L1-KD B16 F10 cell lines) and patient blood	PD-L1	Interaction with PD-1 on T-cells	[100]
Breast cancer cells (MDA-MB-231-luc-D3H2LN cell line)	Adenosine	Binding to the adenosine receptor on T-cell membrane	[101]
Melanoma cells (MV3, WM9, WM902B and WM35 cell lines)	miR-125b-5p	Induction of TAM phenotype in macrophages by targeting the LIPA	[103]
Non-small cell lung cancer cells (A549 cell line)	circATP9A	Induction of the M2 phenotype of TAMs	[104]
Renal cell carcinoma cells (primary 786- O and metastatic ACHN cell lines)	complement C3	Induction of the M2 phenotype of TAMs and recruitment of PMN- MDSCs	[105]

EVs: Extracellular vesicles; PD-L1: programmed death 1 ligand; PD-1: programmed death 1; TAM: tumor-associated macrophage; LIPA: lysosomal acid lipase A; PMN- MDSCs: polymorphonuclear myeloid- derived suppressor cells.

### ECM remodeling

The ECM undergoes extensive remodeling during cancer progression. In this context, malignant cells promote several pathological processes at the matrix level, including: (i) collagen deposition, which provides a supportive scaffold for tumor growth; (ii) molecular cross-linking, which increases ECM stiffness and generates a physical barrier against drugs and immune cell infiltration; (iii) matrix degradation which facilitates cell migration and angiogenesis^[106,107]^. EVs originating from tumor cells take part in this rearrangement by both transporting proteins and nucleic acids involved in matrix deposition/degradation and, more indirectly, promoting CAF formation^[108]^, as summarized in Table 4. Many studies have reported that T-EVs contain high levels of MMPs - especially MMP2 and MMP9 - as well as MMP1, MMP7, and MMP12, together with MMP inducers and kallikreins, all of which contribute to matrix degradation^[109-111]^. MMPs are zinc-dependent endopeptidases responsible for ECM digestion that have been closely associated with cancer progression and dissemination^[112]^. On the other hand, T-EVs can also be rich in components that promote matrix deposition, such as circERC1, which increases collagen I secretion by CAFs and consequently reduces the penetration of drugs and immune cells^[113]^. Another interesting cargo of T-EVs is integrins, which can interact with ECM fibronectin and laminins, inducing cell adhesion and thereby promoting tumor development and colonization^[114]^.

**Table 4:** Molecular mechanisms by which tumor-derived EVs induce extracellular matrix remodeling

**EV source**	**Functional cargo components**	**Mechanism of action**	**Ref.**
Urinary bladder cancer patient urine	MMP7 and MMP12	MMP7 and MMP12 upregulation	[109]
High-metastatic ovarian cancer cells (ES-2 cell line)	MMP1 mRNA	MMP1 upregulation in mesothelial cells	[111]
Pancreatic ductal adenocarcinoma patient tissues	circERC1	Increased collagen I secretion by CAFs	[113]

EVs: Extracellular vesicles; MMP7: matrix metalloproteinase 7; MMP12: matrix metalloproteinase 12; MMP1: matrix metalloproteinase 1; CAFs: cancer-associated fibroblasts.

### Metastasis formation

Cells derived from primary tumors can disseminate through the bloodstream, giving rise to metastases at secondary sites. The metastatic process results from a complex and dynamic interplay involving not only cancer cells but also several TME components. CAFs, TAMs, and the remodeled matrix each contribute to tumor metastatic development in distinct ways^[10]^. Once again, EVs have been observed to be strictly involved in the delivery of signaling molecules promoting migration and invasion [Table 5]. Liu *et al.* reported that TAM-derived EVs could induce EMT in cancer cells, a fundamental step in metastasis that allows cells to detach from primary tumor masses and escape their native environment^[81,115]^. Related to this process, other interesting molecules that can be found in T-EVs include heparanase, which promotes angiogenesis and metastasis^[116]^; ncRNAs such as miR-105, which target the tight junction protein ZO-1 and disrupt vascular endothelial barriers, thereby facilitating cell dissemination^[117]^; and integrins or integrin-like molecules, responsible for cell adhesion and often associated with metastatic sites^[118]^. Moreover, cancer cell- and stroma-derived EVs participate in the development of the so-called “premetastatic niches” (PMNs), microenvironments properly equipped to host metastases, primarily involving lymph nodes as the first sites of metastasis, but also the lungs, liver, and other organs^[119]^. Specifically, T-EVs are enriched in molecules able to activate different signaling pathways related to this process. Chen *et al.* found that TAM-derived EVs containing dedicator of cytokinesis 7 (DOCK7) enhanced colorectal cancer cell migration and invasion through the Ras-related C3 botulinum toxin substrate 1 (RAC1)/ATP-binding cassette sub-family A member 1 (ABCA1) axis^[120]^. In another work, García-Silva *et al.* described how melanoma-secreted EVs could stimulate lymphangiogenesis and metastasis via a nerve growth factor receptor (NGFR)-driven mechanism^[121]^, while Li *et al.* proved that bladder cancer-derived EVs were mediators of a hepatocyte growth factor (HGF)-dependent positive feedback loop between cancer cells and CAFs, promoting lymphatic metastasis^[122]^. Other findings suggested that stroma-derived EVs rich in miR-214 could foster tumor dissemination^[123]^, while breast cancer-released microtubule-associated protein 1 light chain 3 (LC3^+^) EVs can induce PMN formation when activated by heat shock protein 60 (HSP60) through monocyte recruitment and T-cell suppression^[124]^.

**Table 5:** Molecular mechanisms by which tumor-derived EVs foster metastasis formation

**EV source**	**Functional cargo components**	**Mechanism of action**	**Ref.**
THP-1-derived M2-like TAMs	circ_0003137	Promotion of the EMT of glioblastoma cells by targeting the PTBP1/PLOD3 axis	[115]
Breast cancer cells (MDA-MB-231 metastatic cell line)	miR-105	Vascular endothelial barriers destruction by targeting the tight junction protein ZO-1	[117]
Colorectal cancer patient plasma	ITGBL1	Fibroblasts activation through the TNFAIP3/NF-κB signaling axis	[118]
Bone marrow-derived TAMs	DOCK7	Activation of the RAC1/ABCA1 pathway	[120]
Metastatic melanoma cells (SK-MEL-147 cell line)	NGFR	NGFR-driven lymphangiogenesis and tumor cell adhesion	[121]
Bladder cancer patient urine	LINC00665	Activation of a HGF-dependent positive feedback loop between tumor cells and fibroblasts	[122]
miR-214over MEFs and CAFs from PyMT tumors	miR-214	miR-214-mediated crosstalk between tumor and stroma cells favoring tumor dissemination	[123]
Breast cancer cells (4T1 cell line)	LC3 + EVs	Monocyte recruitment and T-cell function suppression	[124]

EVs: Extracellular vesicles; PTBP1/PLOD3: polypyrimidine tract binding protein 1/procollagen-lysine,2-oxoglutarate 5-dioxygenase 3; ZO-1: zonulin 1; ITGBL1: integrin beta-like 1; TNFAIP3/NF-κB: tumor necrosis factor alpha induced protein 3/nuclear factor kappa B; TAMs: tumor-associated macrophages; DOCK7: dedicator of cytokinesis 7; RAC1/ABCA1: Ras-related C3 botulinum toxin substrate 1/ATP-binding cassette transporter A1; NGFR: nerve growth factor receptor; HGF: hepatocyte growth factor; MEFs: mouse embryonic fibroblasts; CAFs: cancer-associated fibroblasts; LC3: microtubule-associated protein 1 light chain 3; EMT: epithelial-mesenchymal transition.

## EV ROLE IN CANCER METABOLISM REPROGRAMMING

Increasing evidence suggests that EVs play a pivotal role in modulating cancer metabolism by altering the metabolic landscape of both tumor and stromal cells^[4]^. This last part of the review aims at summarizing current knowledge on the involvement of EVs in tumor metabolic reprogramming, at the same time highlighting their potential as biomarkers in cancer [Figure 1].

### Glycolytic metabolism

Thanks to their cargo, EVs are continuously secreted by tumor and stromal cells and contribute to glycolytic reprogramming^[125]^. Particularly, EVs can directly or indirectly modulate glycolysis through the release of miRNAs, lncRNAs, circRNAs, and enzymes^[126]^ [Table 6]. In tumors, glycolytic metabolism is reprogrammed to enhance glucose uptake while simultaneously suppressing mitochondrial OXPHOS^[127]^. Wan *et al.* observed that exosomes released by activated hepatic stellate cells (HSCs) can carry both glucose transporter 1 (GLUT1) and pyruvate kinase M2 (PKM2), an enzyme that catalyzes the final step of glycolysis by converting phosphoenolpyruvate to pyruvate, which is subsequently converted into lactate^[128]^. PKM2 is also a known target of hypoxia-inducible factor 1 (HIF-1), a key regulator of the cellular response to hypoxia and an important driver of metabolic reprogramming in cancer^[129]^. Similarly, other reports have demonstrated that EVs isolated from human HCC tissues carry functional enzymes such as lactate dehydrogenase A (LDHA)^[130]^ and glucose-6-phosphate isomerase^[131]^, thereby promoting a glycolytic phenotype in recipient cells. Beyond direct enzyme delivery, T-EVs can also modulate the metabolism and function of immune cells within the TME. For instance, the T-EV uptake by macrophages promotes their polarization toward an M2-like immunosuppressive phenotype, which is characterized by enhanced glycolytic activity^[132]^ through a mechanism dependent on nuclear factor kappa-light-chain-enhancer of activated B cells (NF-κB) signaling^[133]^. Additionally, it has been shown that T-EVs stimulate the expression of inducible nitric oxide synthase (NOS2), an enzyme that inhibits mitochondrial OXPHOS and shifts cellular metabolism toward aerobic glycolysis and lactate production^[134]^. EV-mediated regulation of glucose metabolism is also achieved through ncRNAs. Indeed, T-EVs are known to modulate glycolytic metabolism by releasing nucleic acids such as miRNAs^[135-139]^ and lncRNAs^[140-142]^. Moreover, as shown by Fong *et al.*, T-EVs can reprogram the metabolism of adjacent normal cells by suppressing their glycolytic activity^[143]^. In this way, the tumor is able to enhance its own glucose uptake while inducing metabolic and phenotypic reprogramming in surrounding cells^[114]^. All together, these metabolic changes contribute to the creation of a permissive, immunosuppressive TME that supports tumor progression and immune evasion.

**Table 6:** Molecular mechanisms by which tumor-derived EVs modulate glycolysis

**EV source**	**Functional cargo components**	**Mechanism of action**	**Ref.**
Hepatocellular carcinoma (Hep3B, Bel7404 cell lines)	lncMMPA	M2 polarization of macrophages, miR-548 sponging and glycolysis promotion	[130]
Hepatocellular carcinoma (Hep3B, 97H, LM3 cell lines)	GAPDH, PKM, ALDHA, PGK1, ENO1, TPI1, GPI, PGAM1, PFKP	Supply of key metabolic enzymes involved in glycolysis and gluconeogenesis	[131]
Lung carcinoma (LLC and A549 cell lines)	HMGB-1	Activation of NF-κB signaling pathway, with higher expression of glycolytic enzymes (such as GLUT1, HK2, LDHA), enhanced glycolysis and lactate production and M2-like immunosuppressive polarization	[132]
Breast cancer (MDA-MB-231 cell line)	miR-105	Upregulation of genes coding for key enzymes in glycolysis, glutaminolysis and metabolite transportation.	[135]
Ovarian carcinoma (SKOV cell line)	miR‐21‐5p	Downregulation of *PDHA1,* with limited pyruvate entry into the TCA cycle and enhanced glycolysis	[138]
Osteosarcoma (MG63, U-2OS, MNNG-HOS, Saos-2 cell lines)	HOTAIRM1	Sponging of miR-664b-3p, a key regulator of mTOR pathway	[140]
Melanoma (OL and OL-SD cell lines)	MIR-100hg	Suppression of miR-16-5p and miR-23a-3p, with higher expression of *GLUT1*, *HK2*, *PKM* and *LDHA*	[142]
Breast cancer (MDA-MB-231 cell line)	miR-122	Decreased expression of genes related to the synthesis of GLUT1 and PKM, with increased metastasis and glucose uptake by tumor cells	[143]

EVs: Extracellular vesicles; GAPDH: glyceraldehyde-3-phosphate dehydrogenase; PKM: pyruvate kinase M; ALDHA: aldehyde dehydrogenase; PGK1: phosphoglycerate kinase 1; ENO1: enolase 1; TPI1: triosephosphate isomerase 1; GPI: glycosylphosphatidylinositol; PGAM1: phosphoglycerate mutase 1; PFKP: phosphofructokinase; HMGB-1:high mobility group box 1; NF-κB: nuclear factor kappa B; GLUT1: glucose transporter 1; HK2: hexokinase 2; LDHA: lactate dehydrogenase A; *PDHA1*: pyruvate dehydrogenase E1 subunit alpha 1; TCA: tricarboxylic acid; HOTAIRM1: HOX transcript antisense RNA myeloid-specific 1; mTOR: mechanistic target of rapamycin.

### Lipidic metabolism

EVs contribute to the reprogramming of lipid metabolism both directly, by transporting bioactive molecules such as FAs, cholesterol, eicosanoids, and indirectly, through the delivery of enzymes and regulatory nucleic acids^[144]^ [Table 7]. Even though they are mainly composed of membrane lipids and cholesterol, their lipidic composition can vary according to the cellular origin. As already indicated above, PS is well-known for its pro-tumorigenic activity and has also been proposed as a potential diagnostic marker in cancer^[145]^. Indeed, PS exposure on the outer leaflet of T-EVs and apoptotic cancer cells serves as a potent immunosuppressive signal, promoting the recruitment and polarization of M2-like macrophages and facilitating immune evasion^[23]^. Cholesterol is another key structural component of EVs and contributes to their stability, biogenesis, and functional roles^[146]^. Its abundance in EV membranes has attracted attention not only for its role in EV biology but also for its potential as a diagnostic marker in cancer^[147]^. Regarding this, recent evidence has shown that EVs expressing PD-L1 can hyperactivate lipid metabolism in both human and murine T cells, leading to an increase in cholesterol biosynthesis and lipid droplet accumulation. These metabolic changes contribute to T cell dysfunction and to the activation of immunosuppressive pathways, ultimately favoring tumor immune evasion^[148]^. Beyond structural lipids, EVs can also carry a range of bioactive lipids, including FAs and eicosanoids^[149]^, which are involved in modulating inflammation, cell migration, and tumor progression. In addition, they can deliver various nucleic acids^[150-154]^ which regulate gene expression in recipient cells. Together, these lipid and nucleic acid payloads contribute to membrane remodeling, pro-inflammatory signaling, and enhanced metastatic potential, highlighting the multifaceted role of EVs in tumor progression.

**Table 7:** Molecular mechanisms by which tumor-derived EVs influence lipid metabolism

**EV source**	**Functional cargo components**	**Mechanism of action**	**Ref.**
Leukemia (RBL-2H3 cell line)	Phospholipid scramblase; PLD; PAP1; Phospholipase A2; AA; prostaglandin E2; 15-deoxy- 12,14-prostaglandinJ 2	Signaling pathways modulation by different lipids and enzymes. AA pathway for example promotes tumor progression by modulating the complex interactions between cancer and immune cells within the microenvironment. Numerous prostaglandins, such as E2, are involved in tumor growth, invasion and metastasis	[149]
Breast cancer (MDA-MB-231, MDA-MB-231/Rab27a cell lines)	miR-9-5p	Targeting of INSIG-1 and INSIG-2, involved in cholesterol metabolism, lipogenesis, and glucose homeostasis. Suppression of ATF3, a transcriptional repressor of CH25H. Since CH25H plays a crucial role in tumor immunity, the inhibition of ATF3 by miR-9-5p indirectly promotes a pro-tumorigenic immunosuppressive environment	[151]

EVs: Extracellular vesicles; AA: arachidonic acid; PLD: phospholipase D; PAP1: phosphatidic acid phosphatase type 1; INSIG-1: insulin-induced gene 1 protein; INSIG-2: insulin-induced gene 2 protein; ATF3: activating transcription factor 3; CH25H: cholesterol 25-hydroxylase.

### Amino acid metabolism

T-EVs play a key role in modulating amino acid metabolism in cancer by releasing transporter proteins that allow the TME to uptake essential metabolites [Table 8]. Iraci *et al.* showed that EVs released by neural stem cells (NSCs) are metabolically active and can carry asparaginase-like protein 1 (ASRGL1), an L-asparaginase (L-ASNase)-like protein specific for asparagine that converts the amino acid into aspartate and ammonia^[155]^. Similarly, Liu *et al.* reported that EVs released by pancreatic cells carry L-type amino acid transporter 1 (LAT1), a key amino acid transporter responsible for the uptake of leucine, valine and isoleucine, three amino acids playing a key role in tumor growth and progression^[156]^. As anticipated in the paragraph “*Glutamine metabolism* “, Gln represents a fundamental amino acid for tumor cells, serving as a major source of carbon and nitrogen for the biosynthesis of nucleotides and lipids. Moreover, it serves as an alternative energy substrate under glucose-deprived conditions and contributes to the synthesis of glutathione, a key molecule responsible for maintaining redox homeostasis. Glutamine is internalized by cells mainly through the alanine, serine, cysteine-preferring transporter 2 (ASCT2)^[157]^. Notably, EVs can modulate this process by transferring and releasing ASCT2, thereby enhancing glutamine uptake and its subsequent mitochondrial metabolism^[158,159]^. Recently, it has been shown that in gastric^[160]^ and lung cancer^[161]^, EVs can transport the oncoprotein cellular myelocytomatosis oncogene (c-Myc), which positively regulates *GLS-1* (*glutaminase-1*) expression, an enzyme that converts glutamine into glutamate. This leads to enhanced glutaminolysis, which sustains tumor cell proliferation and provides biosynthetic precursors essential for rapid growth. Moreover, c-Myc represses miR-23a and miR-23b, two miRNAs that normally inhibit glutamine metabolism^[137,162]^. By doing this, EVs are able to enhance glutamine metabolism and drive tumors toward a glutamine-addiction phenotype. Meanwhile, EVs are known to carry nucleic acids that can modulate glutamine metabolism. Representative examples include HOTAIR and NT5E^[163,164]^, a lncRNA and a circRNA, respectively, which have been identified in lung cancer and glioma. These two nucleic acids can silence miR-153 and miR-203, two miRNAs that negatively regulate *GLS-1* activity^[165,166]^ and, consequently, affect GLS activity. By suppressing these miRNAs, Gln metabolism is enhanced, allowing the tumor to grow and proliferate.

**Table 8:** Molecular mechanisms by which tumor-derived EVs affect amino acid metabolism

**EV source**	**Functional cargo components**	**Mechanism of action**	**Ref.**
Pancreatic cancer (T3M-4 cell line)	LAT1	Transport of LAT1, a protein responsible for the transport of neutral amino acids such as leucine, isoleucine, valine, phenylalanine and tyrosine	[156]
Umbilical vein endothelial cells (HUVEC cell line)	SLC1A5	Enhanced glutamine metabolism and tumor invasion, migration and growth via EGFR/SRC/YAP1/GPX4 signaling cascade	[159]
Lung carcinoma (A549, LT73 cell lines)	c-Myc	Suppression of miR-23a and miR-23b, known to decrease glutamine metabolism by binding to the 3′ UTR region of *GLS-1.* Upregulation of SLC7A5 and ASCT2, with increased glutamine levels in the TME	[161,162]
Glioma (U87, U251 cell lines)	NT5E	Sponging of miR-153, known for being involved in the suppression of tumor growth and development by binding *GLS-1*	[163,166]
Lung carcinoma (Serum)	HOTAIR	Sponging of miR-203, a tumor-suppressive miRNA downregulating glutaminase expression	[164,165]

EVs: Extracellular vesicles; LAT1: L-type amino acid transporter 1; SLC1A5: solute carrier family 1 member 5; EGFR/SRC/YAP1/GPX4: epidermal growth factor receptor/SRC proto-oncogene non-receptor tyrosine kinase/yes-associated protein 1/glutathione peroxidase 4; *GLS-1*: glutaminase 1; SLC7A5: solute carrier family 7 member 5; ASCT2: alanine/serine/cysteine-preferring transporter 2; TME: tumor microenvironment; NT5E: 5’-nucleotidase ecto; HOTAIR: HOX transcript antisense RNA.

Tumor metabolism is highly coordinated and interconnected^[167]^, and EVs actively contribute to this vital metabolic crosstalk^[168]^ by carrying a very heterogeneous cargo that includes proteins, lipids, and miRNAs. Importantly, EVs do not simply modulate isolated pathways; rather, they orchestrate a synergistic and flexible metabolic network that sustains proliferation, immune suppression and therapy resistance. For instance, the enhanced glycolytic flux driven by EV-mediated delivery of GLUT1, PKM2, or LDHA increases lactate secretion. Lactate has been detected in several types of EVs^[169]^ and contributes not only to acidification of the TME but also enhances tumor resistance by activating DNA-repair mechanisms^[170]^. Moreover, lactate is transported by EVs and taken up by recipient tumor cells, where it can be oxidized again within the cytosol and converted back into pyruvate^[126]^. Glucose-derived pyruvate is then converted to acetyl-CoA and enters the TCA cycle to be transformed to citrate which is exported from the mitochondria to the cytosol by the citrate carrier (CIC), serving as the essential feedstock for *de novo* FA synthesis, which satisfies the elevated lipid requirement for membrane production. Here, again, EVs play a key role by removing citrate from tumor cells, whose intracellular accumulation is metabolically unfavorable, as it exerts negative feedback on glycolysis, TCA and insulin-like growth factor 1 receptor (IGF-1R) pathways^[171]^, and redistributing it within the TME, where it can be reutilized as a carbon substrate and again enter the TCA cycle^[172]^. Moreover, as previously mentioned, EVs can mediate the intercellular transfer of amino acid transporters, such as ASCT2^[173]^ and LAT1^[156]^, thereby enhancing amino acid uptake and metabolic adaptation in cancer cells. Glucose and amino acid metabolism are tightly interconnected. A typical example is serine and PKM2: serine acts as an allosteric activator of PKM2^[174]^, which in turn promotes *de novo* serine synthesis^[175]^. In addition, as previously mentioned, glutamine serves as a major anaplerotic substrate, replenishing TCA cycle intermediates and sustaining biosynthetic processes, including nucleotide, amino acid, and lipid synthesis^[27]^. By doing so, tumor cells can maintain mitochondrial function and redox balance^[28]^, thereby supporting proliferation even when glucose is available. In conclusion, metabolic reprogramming in cancer cells is characterized by the concurrent upregulation of glycolysis and glutaminolysis, which cooperatively supply intermediates for anabolic processes^[176]^. Together, these mechanisms highlight how cancer cells exploit metabolic plasticity to dynamically redistribute carbon sources, thereby sustaining anabolic signaling and proliferation under fluctuating nutrient conditions within the TME.

## DISCUSSION AND CONCLUSIONS

TME plays an important role in supporting tumor growth. Tumor cells can indeed affect TME components, thereby altering their behavior pathologically. Interestingly, tumor-derived EVs are involved in the transfer of neoplastic traits, contributing to tumor development and dissemination. In particular, it has been demonstrated that, due to their cancer-related cargo, they can mediate key processes, including angiogenesis, fibroblast transformation into CAFs, immune evasion, ECM remodeling, metastasis promotion, and metabolic reprogramming. These effects are mainly driven by the release of specific molecules, primarily proteins and nucleic acids, that activate distinct signaling pathways in recipient cells. However, many of these mechanisms remain only partially understood, and a deeper investigation would be crucial not only for a more comprehensive characterization of the TME, but also for the identification of novel diagnostic and therapeutic targets.

Indeed, despite significant progress, several unresolved questions remain regarding EV-mediated modulation of the TME and cancer metabolism. First, the extent to which EV cargo reflects functional selectivity *vs*. stochastic packaging is still debated^[177]^, and the molecular determinants guiding cargo loading remain only partially defined^[178]^. Second, technical challenges, including the heterogeneity of EV isolation methods, limited *in vivo* tracking tools, and the difficulty in unambiguously distinguishing EV subtypes, continue to hinder the translation of experimental findings into clinical applications. These issues are not restricted to metabolic studies but represent methodological limitations across the entire EV research field. Indeed, even with the implementation of MISEV (Minimal Information for Studies of Extracellular Vesicles) guidelines, substantial variability persists in EV purification strategies, ranging from differential ultracentrifugation to size-exclusion chromatography and immunoaffinity capture, which can yield vesicle populations with distinct biophysical properties and molecular profiles^[52]^. Furthermore, current *in vivo* EV-tracking approaches, including fluorescent labeling, bioluminescent reporters and metabolic tagging, lack sufficient resolution to discriminate EV sources or to reliably quantify biodistribution, thereby constraining our understanding of EV dynamics within complex tumor ecosystems^[179]^. In addition, defining the respective contributions of stromal-derived *vs*. tumor-derived EVs remains challenging due to the absence of robust lineage-specific markers and the frequent overlap in size and composition among different EV subtypes^[180]^. This uncertainty becomes even more pronounced under nutrient-restricted or therapy-induced stress conditions, where both tumor and stromal cells markedly alter EV secretion rates and cargo composition^[181]^. As a result, disentangling the reciprocal metabolic influences mediated by these heterogeneous vesicle populations remains an unresolved barrier for mechanistic and translational studies across the EV field. Overcoming these barriers will be critical for transforming the growing mechanistic understanding of EV biology into clinically meaningful diagnostic tools and therapeutic strategies. Technical challenges, including heterogeneity of isolation methods, limited *in vivo* tracking tools, and difficulty distinguishing EV subtypes, hinder the translation of experimental findings into clinical applications. Moreover, the contribution of stromal-derived *vs*. tumor-derived EVs in shaping metabolic symbiosis in the TME is not fully understood, particularly in nutrient-restricted or therapy-induced stress conditions.

Future research should aim to develop standardized EV characterization pipelines (in accordance with MISEV guidelines), advanced *in vivo* imaging strategies, and systems-biology approaches capable of integrating metabolic fluxes with EV secretion dynamics. Additionally, defining whether specific EV metabolic signatures can serve as predictive biomarkers or therapeutic targets remains an important unmet need. These aspects will be crucial to improve our mechanistic understanding and to support the design of EV-based interventions in oncology.

## DECLARATIONS

### Copyright

© The Author(s) 2026.
